# A Comparison of Physiological Signal Analysis Techniques and Classifiers for Automatic Emotional Evaluation of Audiovisual Contents

**DOI:** 10.3389/fncom.2016.00074

**Published:** 2016-07-15

**Authors:** Adrián Colomer Granero, Félix Fuentes-Hurtado, Valery Naranjo Ornedo, Jaime Guixeres Provinciale, Jose M. Ausín, Mariano Alcañiz Raya

**Affiliations:** Instituto de Investigación e Innovación en Bioingeniería, Universitat Politècnica de ValènciaValencia, Spain

**Keywords:** audiovisual content evaluation, effectiveness, electroencephalography (EEG), electrocardiography (ECG), galvanic skin response (GSR), respiration, feature extraction, advanced classifiers

## Abstract

This work focuses on finding the most discriminatory or representative features that allow to classify commercials according to negative, neutral and positive effectiveness based on the Ace Score index. For this purpose, an experiment involving forty-seven participants was carried out. In this experiment electroencephalography (EEG), electrocardiography (ECG), Galvanic Skin Response (GSR) and respiration data were acquired while subjects were watching a 30-min audiovisual content. This content was composed by a submarine documentary and nine commercials (one of them the ad under evaluation). After the signal pre-processing, four sets of features were extracted from the physiological signals using different state-of-the-art metrics. These features computed in time and frequency domains are the inputs to several basic and advanced classifiers. An average of 89.76% of the instances was correctly classified according to the Ace Score index. The best results were obtained by a classifier consisting of a combination between AdaBoost and Random Forest with automatic selection of features. The selected features were those extracted from GSR and HRV signals. These results are promising in the audiovisual content evaluation field by means of physiological signal processing.

## 1. Introduction

Estimation of emotional states is a powerful tool in the marketing field. Efficient monitoring of human emotional states may provide important and useful information for marketing purposes (Frantzidis et al., 2010a). Such monitoring could follow either subjective or objective methods. Subjective methods (psychology-oriented approach) are based on qualitative behavior assessment or by means of questionnaires and interviews, whilst objective methods (neuropsychology-oriented approach) consist on monitoring and analyzing the subject biosignals (Frantzidis et al., 2010a).

It is now recognized that making use of standard marketing techniques, such as depth interviews or focus groups, in which customers are exposed to the product in advance of its massive launch or afterwards, provides biased answers due to the respondents cognitive processes activating during the interview and by the influence that the interviewer may have on their recalls (Vecchiato et al., 2014). Furthermore, people are not able to (or might not want) fully express their preferences when they are explicitly asked (Vecchiato et al., 2011a). Therefore, marketing researchers prefer to complement traditional methods with the use of biosignals.

To follow the objective approach, different features of either positive or negative emotions can be extracted from physiological signals, such as electrocardiography (ECG), electroencephalography (EEG), galvanic skin response (GSR) or the breathing response (Frantzidis et al., 2010a). This techniques allow to assess human emotions in terms of it is able to reveal information that is unobtainable employing traditional methods (Vecchiato et al., 2014).

Electroencephalography and the magnetoencephalography (MEG) allow to record on a millisecond basis the brain activity during the exposition to relevant marketing stimuli. However, such imaging brain techniques present one difficulty: the recorded cerebral activity is mainly generated on the cortical structures of the brain. It is almost impossible to acquire the electromagnetic activity yield by deep structures which are often associated with the generation of emotional processing in humans with EEG or MEG sensors. To overcome this problem, high-resolution EEG technology has been developed to enhance the poor spatial information content on the EEG activity. With this technology, brain activity can be detected with a spatial resolution of a squared centimeter on a milliseconds basis, but only in the cerebral cortex.

Furthermore, autonomic activity such as Heart Rate (HR) and Galvanic Skin Response (GSR) are also able to assess the internal emotional state of the subject (Christoforou et al., 2015; Ohme et al., 2011). GSR activity is actually a sensitive and convenient way of measuring indexing changes in sympathetic arousal associated with emotion, cognition and attention (Critchley, 2002). Lang et al. (1993) discovered that the mean value of GSR is related to the level of arousal. Blood pressure and Heart Rate Variability (HRV) also correlate with emotions, since stress may increase blood pressure. Pleasantness of stimuli can increase peak heart rate response, and HRV decreases with fear, sadness and happiness (Soleymani et al., 2008). Respiration has proven to be an adequate emotional indicator. It is possible to distinguish relaxation (slow respiration) and anger or fear (irregular rhythm, quick variations and cessation of respiration). It is possible as well to detect laughing because it introduces high-frequency fluctuations to the HRV signal (Appelhans and Luecken, 2006).

Different authors have attempted to classify audiovisual content attending to elicited emotions in watchers by means of analyzing physiological signals. Features are extracted from the signals and classified with different data mining algorithms, such as Mahalanobis Distance-based (MD) classifier, Support Vector Machines (SVMs) or C4.5 decision tree (Frantzidis et al., 2010a,b).

Another approach is to classify audiovisual content attending to extracted characteristics from audio or both audio and video tracks (Wang et al., 2009). In both cases the same algorithms are applied to the extracted features: Hidden Markov Models (HMM), Dynamic Bayesian Networks (DBM), Gaussian Mixture Models (GMM) and fuzzy methods (Teixeira et al., 2012).

In this paper, we aim to build a robust method to automatically find the most discriminating features that allow to classify a commercial ad in three classes (positive, neutral or negative) based on the physiological response of the subject. These three classes tell the ad's power based on the ACE score index to engage the person watching it. To achieve this, we use different state-of-the-art machine learning techniques for extracting features and classifying the ads watched by the subject.

This is the basis for future studies trying to find the ad effectiveness segmenting by gender, age, geographic location, etc. which will help companies to better develop effective ads focused on a specific audience.

In the remaining part of the paper, the experimental design and the preprocessing steps, along with theoretical steps regarding the feature extraction procedure and classifiers are reported in Section 2, followed by the detailed presentation of the results in Section 3. Finally, the discussion of the results can be found in the last section.

## 2. Materials and methods

### 2.1. Material

Our sample consisted of forty-seven voluntary and healthy subjects (22 males and 25 females), aged between 25 ± 5 years old. However, EEG data from twelve subjects, ECG and respiration data from four subjects and GSR data from three subjects were removed due to corrupted data. The corrupted data produce standard deviation higher than the average value. All participants had normal or corrected-to-normal vision and hearing and they had not participated in a brain study before. The study was approved by the Institutional Review Board of Universitat Politècnica de València with written informed consent from all subjects. All subjects gave written informed consent in accordance with the Declaration of Helsinki.

In order to carry out a base study, firstly we selected eight commercials showed on the Super Bowl 2013. These eight ads were selected according to the Ace Score: positive, neutral and negative ads were chosen. Ace Score is the measure of ad creative effectiveness based on viewer reaction to national TV ads. Respondents are randomly selected and representative of the U.S. TV viewing audience. The results are presented on a scale of 1-950. The ad under analysis “The Date” of Heineken completes our selection. Table 1 shows a summary of the selected commercials and the classification following the Ace Score index.

**Table 1:** **Commercials involved in this study and grouped taking into account the Ace Score index**.

**Commercial**	**Ace Score**	**Group**
Budweiser (“Brotherhood”)	665	Positive
Coke (“Security Camera”)	641	Positive
Doritos (“Goat 4 Sale”)	626	Positive
Hyundai (“Stuck”)	611	Positive
Audi (“Bravery”)	394	Neutral
Calvin Klein (“Concept”)	362	Neutral
“Pub Loo Shocker”	210	Negative
“Carmel Limo”	167	Negative
Heineken (“The Date”)	Non-evaluated	-

**Figure 1:**
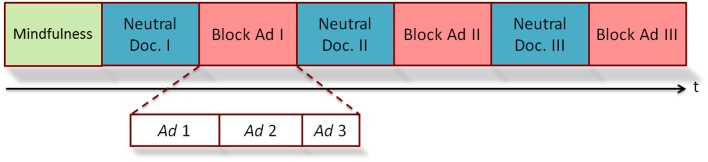
**Diagram about the experimental design**.

The procedure of the experimental task consisted in observing a 30-min documentary about the submarine world in which three blocks of Super Bowl ads were inserted: the first one after 7 min from the beginning of the documentary, the second one in the middle and the last one at the end of the trial. Each of these blocks was formed by three commercials (Figure 1). This audiovisual content were randomly distributed to remove the factor “sequence” as possible confounding effect in the later analysis.

Two hours after the experiment, users were interviewed using an online test. In this test different frames of proposed ads were presented. The user must connect the frames presented with the correct ad brands. The purpose of this interview was to know which ads were remembered and forgotten by the subjects.

### 2.2. Signal recording

#### 2.2.1. Cerebral recording

**Figure 2:**
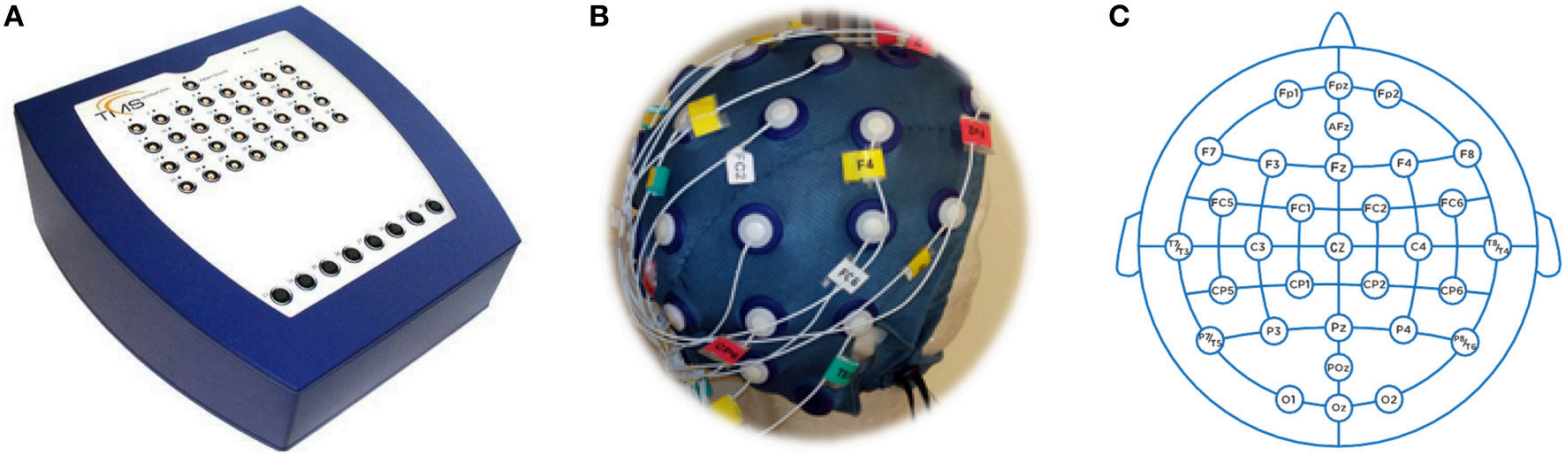
**EEG instrument**. **(A)** Amplifier, **(B)** Cap, **(C)** Distribution.

The cerebral activity was recorded using an instrument developed by Twente Medical Systems International (TMSI from Oldenzaal, The Netherlands). This device consists in an amplification and a digitalization stage. The amplifier (model REFA 40-channels, Figure 2A) is composed by 32 unipolar, 4 bipolar, 4 auxiliary and 8 digital inputs. The TMSI instrument allows the synchronization, via hardware, from its inputs.

All subjects were comfortably seated on a reclining chair 60 centimeters away from the screen. The screen used was a 23 inches Full HD resolution (1920 × 1080 pixels). EEG activity was collected at a sampling rate of 256 Hz while impedances kept below 5kΩ. For the experiment, we used thirty electrodes (Figure 2B) and a bracelet ground located on the opposite wrist to the habitual subject hand. The montage followed the International 10–20 system (Jasper, 1958; Figure 2C).

#### 2.2.2. Autonomic recordings

**Figure 3:**
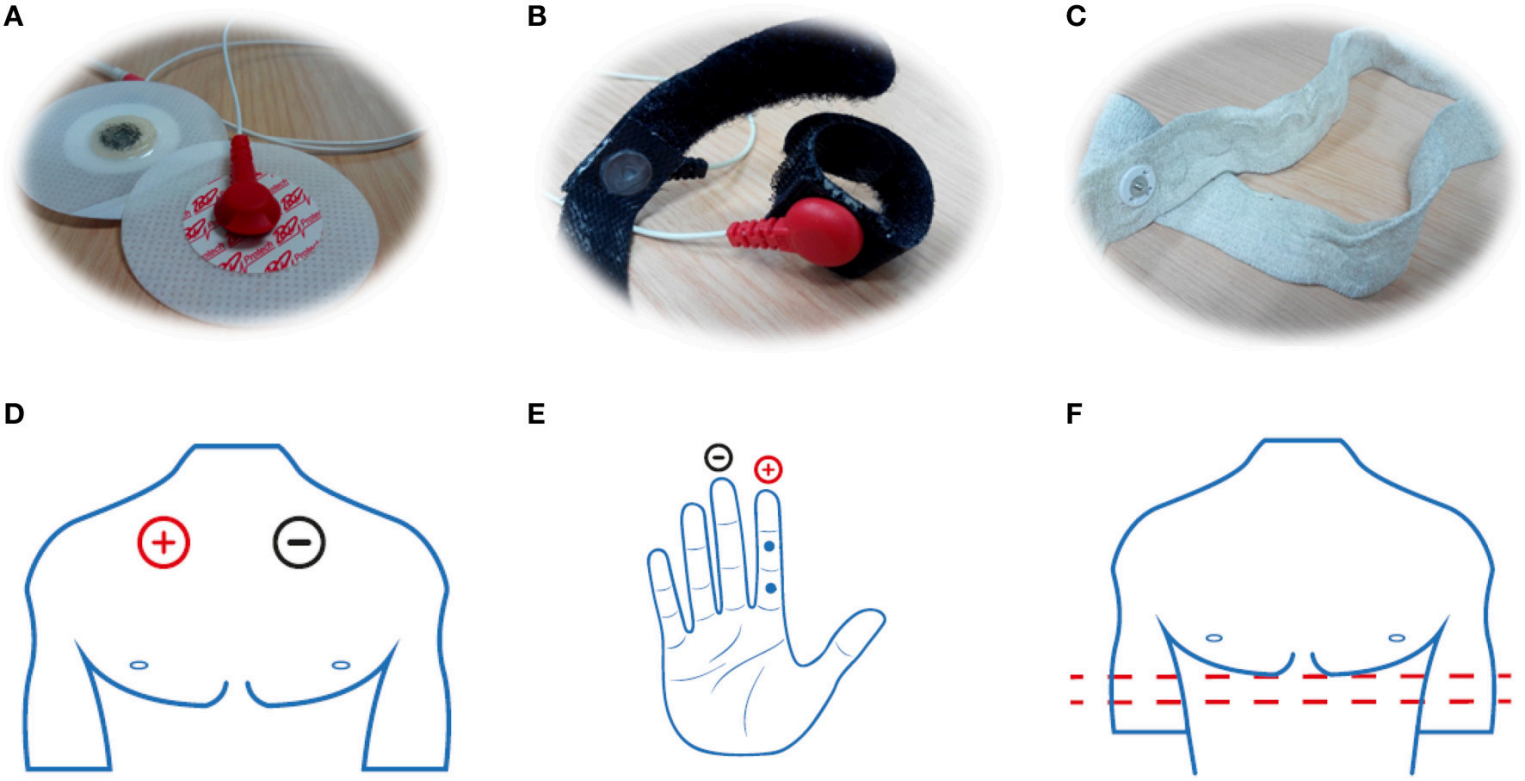
**(A)** EEG, **(B)** GSR, and **(C)** RSP sensors and their respective locations **(D–F)**.

Using the TMSI instrument and software solution for neuroscience experiments (Neurolab from Bitbrain, Spain) it is possible to acquire synchronized biosignals according to the audiovisual content under evaluation. By means of two bipolar inputs, the cardiac activity of each participant can be registered. Two disposable electrodes (Figure 3A) are placed on the upper chest. The first one, the electrode plugged into positive terminal of the amplifier, is placed below of the right clavicle and the other one, the electrode plugged into negative terminal of the amplifier, is placed below of the left clavicle (Figure 3D).

To measure the skin ability to transmit electrical currents incremented due to sweating and organism changes, a galvanic response sensor is used. This sensor consists in two cloth strips (with velcro) in which there is an electrode sewn (Figure 3B). The strips are placed on the fingers of the non-dominant hand. Specifically, the electrode belonging to the positive terminal is placed on the middle or proximal phalanx of the index finger. In addition, the electrode belonging to the negative terminal is placed on the middle or proximal phalanx of the middle finger (Figure 3E). This sensor is plugged into the auxiliary channels of the amplifier.

It is possible to plug a rubber band consisting of two electrodes into one of the eight auxiliary channels in order to measure the breathing (Figure 3C). This rubber band is placed on the bottom of the rib cage (Figure 3F). The sensors measure the rubber band deformation produced by the inhalation and exhalation phenomena.

### 2.3. Signal preprocessing

#### 2.3.1. Cerebral signal

The baseline of EEG traces is removed by mean subtraction and the output dataset is band pass (0.5–40 Hz) filtered. Then, the corrupted data channels are rejected and interpolated from the neighboring electrodes. A corrupted data channel is identified computing the fourth standardized moment (kurtosis) along the signal of each electrode. The kurtosis is defined as:

(1) $$K(x)=\frac{\mu_{4}}{\sigma^{4}}=\frac{E[{\left(x-\mu \right)}^{4}]}{E{\left[{\left(x-\mu \right)}^{2}\right]}^{2}}$$

where μ_4_ is the fourth moment about the mean, σ is the standard deviation and *E*[*x*] is the expected value of the signal *x*. Moreover, a channel is also classified as corrupted if the registered EEG signal is flatter than 10% of the total duration of the experiment.

Reference events are integrated into the data structure in order to segment the EEG signal in epochs of one second. The intra-channel kurtosis level of each epoch is computed in order to reject the epochs highly damaged by the noise.

**Figure 4:**
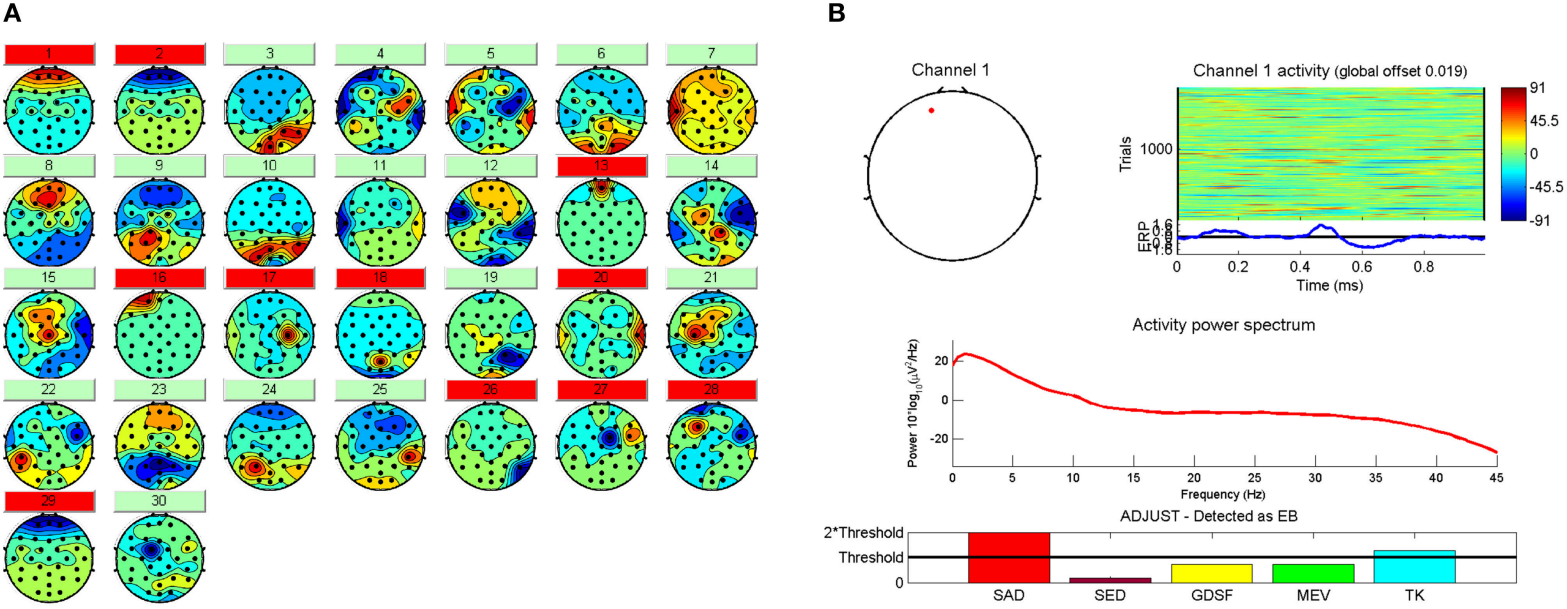
**(A)** The 30 IC's with the artifact components marked in red to be rejected. **(B)** Spatial and temporal features and the frequency spectrum related to the first component marked as artifact by ADJUST.

In the next step, Independent Component Analysis (ICA) (Hyvärinen and Oja, 2000) is applied by means of *runica* algorithm to detect and remove components due to eye movements, blinks and muscular artifacts. Thirty source signals are obtained (one per electrode). Then, an automatic and embedded Matlab method (ADJUST) (Mognon et al., 2011) is used to discriminate the artifact components from EEG signals by combining stereotyped artifact-specific spatial and temporal features. Components whose features accomplish certain criteria are marked to reject (Figure 4A). See Mognon et al. (2011) for detailed explanation of ADJUST. In Figure 4B the spacial and temporal features extracted by ADJUST algorithm of a typical eye blink can be seen.

In the automatic process of artifact component identification, ADJUST presents several true negatives, in other words, there exists components which are composed by a lot of physiological noise and a little useful information (brain activity) that the algorithm does not mark to be rejected. For this reason, a trained expert analyses manually the features of each component (the topographic distribution of the signal, the frequency response, the temporal and spatial features extracted by ADJUST, etc.) in order to discover the remained artifact components. The final objective in the preprocessing stage is to guarantee a compromise between brain activity signal removal and artifact remaining.

**Figure 5:**
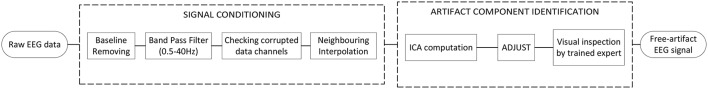
**Architecture of the EEG preprocessing stage**.

Figure 5 shows a diagram of the whole processing stage. After this, the EEG signal is free of artifacts and it can be analyzed in the next stage using the feature extraction metrics presented in Section 2.4.

In order to develop the proposed preprocessing algorithm, EEGLAB (Delorme and Makeig, 2004) and ADJUST (Mognon et al., 2011) libraries were used.

#### 2.3.2. Autonomic signals

To analyze the electrocardiogram signal, the QRS complex detection is required, so the preprocessing of the cardiac signal is a very important step. First, the ECG signal is high-pass filtered in order to correct the baseline problems as baseline wander caused by the effects of electrode impedance, the respiration or body movements. A FIR filter (with cut off frequency of 0.5 Hz) is used for this purpose in order to avoid the phase distortion produced by a IIR filter, which would modify the wave morphology. In addition, the signal DC component is eliminated subtracting the mean. The next step is to apply a Notch filter in order to avoid the power line interference (the interfering frequency is *w*_0_ = 50*Hz*). Muscle noise cause severe problems as low-amplitude waveform being obstructed. To eliminate this noise a low-pass filtered (with a cut off frequency ranged from 60 to 70 Hz) is applied.

Regarding the GSR and RSP preprocessing, a morphological filter is employed to remove the signal ripple in order to facilitate the local maxima detection. This low-pass filter allows the elimination of the muscle noise (high frequencies) in order to detect more accurately the sweating peaks (into the GSR signal) and the inhalation/exhalation peaks (into the RSP signal).

### 2.4. Feature extraction

#### 2.4.1. EEG

##### Global field power

The recorded signal obtained directly from the scalp shows intra-cranial synchronous activation of many neurons. To quantify the amount of cerebral activity, the Global Field Power (GFP) (Lehmann and Skrandies, 1980) was employed using Equation (2).

(2) $$GFP=\sqrt{\frac{\sum_{i\;=\;1}^{N_{e}}\sum_{j\;=\;1}^{N_{e}}{\left(u_{i}-u_{j}\right)}^{2}}{N_{e}}}$$

where *u*_*i*_ is the potential at the electrode *i* (over time), *u*_*j*_ is the potential at the electrode *j* (over time) and *N*_*e*_ is the total number of electrodes employed to compute the GFP.

Frontal areas are the cerebral locations mainly involved in the memorization and pleasantness phenomena (Vecchiato et al., 2010). Thus, the electrodes Fp1, Fpz, Fp2, F7, F3, Fz, F4, F8, Fc5, Fc1, Fc2, and Fc6 were taken into account in the calculation. A GFP signal was then calculated for each frequency band considered in the experiment: δ (1–3 Hz), θ (4–7 Hz), α (8–12 Hz), β (13–24 Hz), β extended (25–40 Hz), and γ (25–100 Hz).

The blocks of neutral documentary (one before each ad block) are baseline periods taken as a reference. The purpose of these blocks is to be able to register the basal cerebral activity to remove phenomena as fatigue or lack of concentration. GFP normalization according to baseline periods provides the *Zscore* index computed as:

(3) $$Zscore=\frac{GFP_{i}-\bar{GFP_{B}}}{\sigma (GFP_{B})}$$

where *GFP*_*i*_ is the Global Field Power during the ad under analysis, *GFP*_*B*_ is the Global Field Power during a period of 2-min of the neutral documentary previous to the block of ads where is the ad under analysis located (Figure 1).

For each stimulus the input to the different classifiers is the time-average value of the GFP, *Zscore* and log(*Zscore*) in each frequency band.

##### Interest index (II)

The interest index allows the commercial assessment in specific time periods in Theta and Beta bands (Vecchiato et al., 2010). For each ad and subject the most significant peaks for *Zscore* variable were obtained, considering a peak all values that exceeds the threshold of *Zscore* ≥ 3, associated with a *p* < 0.05 in the Gaussian curve fitted over *Zscore* distribution (averaged for all participants).

In this way, two parameters were calculated: the number of peaks during the total duration of a particular commercial (*PN*_*total*_) and the number of peaks during the brand exposition periods of a particular commercial (*PN*_*brand*_). The interest index is computed for each ad and subject as:

(4) $$II=\frac{PN_{brand}}{PN_{total}}$$

The input to the different classifiers is the percentage of interest for each commercial.

##### Memorization index (MI)

This index allows to measure the capacity of each stimulus to be remembered (Vecchiato et al., 2011b). First, the GFP in theta and alpha bands (associated with human memorization process Vecchiato et al., 2011b) are normalized following:

(5) $$MI=\frac{GFP_{i}}{\sum_{i\;=\;1}^{M}GFP_{i}}$$

where *GFP*_*i*_ is the Global Field Power along the duration of the stimulus under analysis *i* and *M* is the number of temporal samples.

In order to extract the memorization index, an on-line survey is carried out following the method used in Vecchiato et al. (2011c). Two hours after the experiment ends each participant has to complete the on-line test designed by specialists in psychology. In this test different frames of each commercial are presented and the subject must answer some questions about these frames. By means of this test we can check the stimuli remembered and forgotten for each participant.

For each commercial, the population was segmented in two groups. Subjects who remembered the ad were included in the “remember group” and those who forgot it were included in the “forget group”.

**Figure 6:**
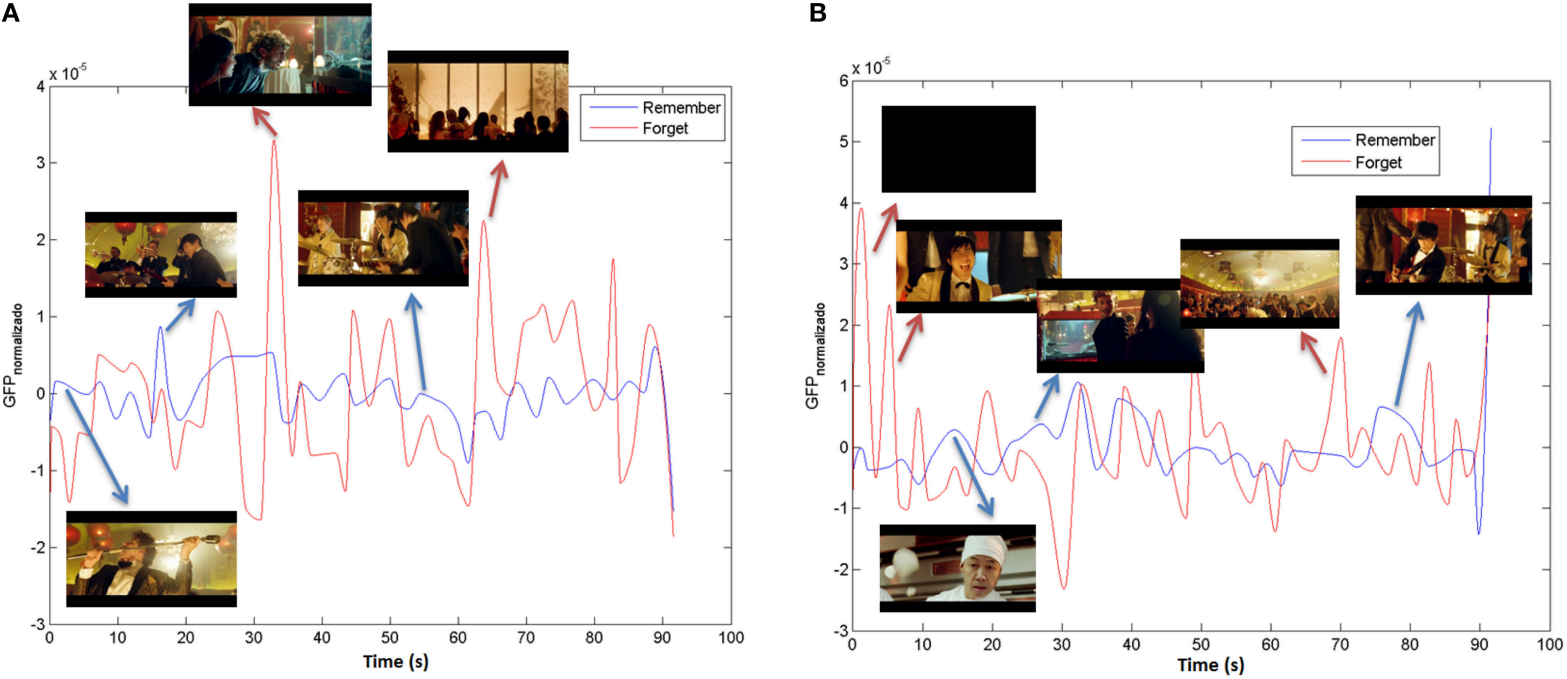
**Memorization index in (A) Theta and (B) Alpha bands for “The Date”**.

It is possible to compute the *GFP*_*Remember*_ as the Global Field Power average of participants that belong to “remember group” for each stimuli. In the same way, a *GFP*_*Forget*_ can be extracted taking into account the “forget group.” Finally the remember and forget indexes are computed by means of a cubic smoothing from the *GFP*_*Remember*_ and the *GFP*_*Forget*_ in order to extract the signal envelope. In Figure 6 the MI in theta and alpha bands for the stimulus under study (“The Date”) can be observed.

The input to the different classifiers is the time-average value of the remember and forget indexes for each stimulus.

##### Pleasantness index (PI)

The pleasantness index is a continuous metric along the time that provides information about the moments of the audiovisual content that are pleasing to the participants (Vecchiato et al., 2013). The cerebral activity registered by the left-frontal electrodes is compared with the cerebral activity registered by the right-frontal electrodes, so the Global Field Power in the Theta and Alpha bands are computed employing asymmetric pairs of electrodes obtaining *GFP*_*Left*_ and *GFP*_*Right*_ for each participant and stimulus.

From the on-line survey explained in the previous section, it is possible to know the participants pleasure about each audiovisual content under study. Using this information, the population is segmented in two groups: “Like” and “Dislike.” The *GFP*_*Left*_ and *GFP*_*Right*_ for each group is obtained by means of the Global Field Power (along the stimulus under analysis) average.

It is possible to extract the pleasantness index for each group as:

(6) $$PI=GFP_{Right}(L/D)-GFP_{Left}(L/D),$$

**Figure 7:**
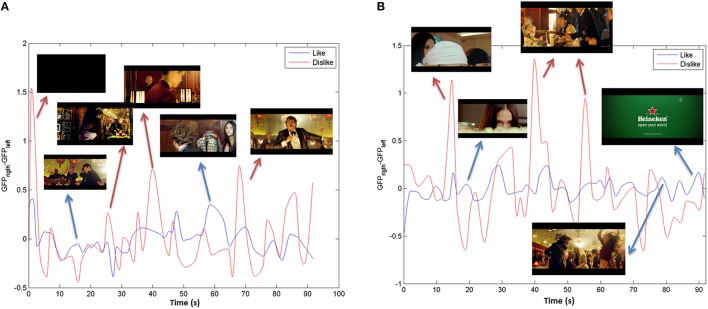
**Pleasantness index in (A) Theta and (B) Alpha bands for “The Date**.”

Finally, like and dislike pleasantness indexes are computed by means of a cubic smoothing from the *PI*_*Like*_ and the *PI*_*Dislike*_ in order to extract the signal envelope. In Figure 7 the PI in theta and alpha bands for the stimulus under study (“The Date”) can be observed.

The input to the different classifiers is the time-average value of the like and dislike indexes for each stimulus.

##### Power spectral density (PSD)

The amount of power in each frequency band and electrode was computed by means of the Welch periodogram (Welch, 1967). In particular, the average of PSD is computed as:

(7) $$\bar{PSD_{c,s}}=\frac{1}{N_{w}}\sum_{i\;=\;0}^{N_{w}-1}P_{x_{i}}$$

where *N*_*w*_ is the number of windows along the signal of the channel *c* in the stimulus *s* and *P*_*x*_*i*__ is the periodogram for the *ith* window calculated as:

(8) $$P_{x_{i}}=\frac{1}{N}|FFT_{N,x_{i}}{|}^{2}≜\frac{1}{N}{\left|\sum_{n\;=\;0}^{N-1}x_{i}(n)e^{-j2\pi nk/N}\right|}^{2}$$

where *N* is the number of points to compute the *FFT*.

The window size used to compute the Welch periodogram was 128 samples corresponding to half second of the EEG signal and the percentage of overlapping was 50%. The input to the different classifiers are 6 (δ, θ, α, β, β extended and γ) × *N*_*pe*_ features for each stimulus being *N*_*pe*_ the number of asymmetric electrodes pairs used in the experiment.

#### 2.4.2. ECG

After ECG preprocessing, the QRS is detected, specifically the R wave, by means of Pan-Tompkins' algorithm (Pan and Tompkins, 1985). The analysis of variations in the instantaneous heart rate time series using the beat-to-beat RR-intervals (the RR tachogram) is known as Heart Rate Variability (HRV) analysis (American Heart Association, 1996). The balance between the effects of the sympathetic and parasympathetic systems, the two opposite acting branches of the autonomic nervous system, is referred to as the sympathovagal balance and is believed to be reflected in the beat-to-beat changes of the cardiac cycle (Kamath, 1991). The HRV analysis is based on feature extraction from the tachogram signal in four domains: time, frequency, time-frequency and non-linear analysis. All signals were reviewed manually by an expert after the automatic R wave detection to avoid the existence of false positives or false negatives and with the aim of delete extremely noisy sections which could not be analyzed. In this manner, the non-existence of artifacts which could alter the signal is assured.

Some parameters extracted in the time domain used in this study were: the maximum (*maxRR*) and minimum (*minRR*), the average (*meanRR*), the median (*medianRR*) and the standard deviation between RR intervals (*SDRR*), the standard deviation from the RR average interval in time-windows (*SDARR*), the square root of the sum of the successive differences between adjacent RR intervals (*RMSSD*), the number of successive RR pairs having a difference less than 50 ms (*RR*_50_) and the ratio between the *RR*_50_ and the total RRs (*pRR*_50_).

The Power Spectral Density (PSD) analysis provides information about the amount of power in several frequency ranges of the tachogram signal. The analysis in the frequency domain was carried out in four frequency bands: ULF(0–0.033 Hz), VLF(0.033–0.04 Hz), LF(0.04–0.15 Hz), and HF(0.15–0.4 Hz) bands. For this work, the ULF and VLF bands are ignored because these frequency bands are only important in 24-h registers. The amount of power in each band is obtained integrating the PSD signal between the bounds of the frequency bands. The power metrics are presented in absolute values (*aLF, aHF*), normalized to the total energy (*nLF, nHF*) or in a percentage value of the total energy (*pLF, pHF*). The power ratio between the LF and HF band provides information about the sympathetic/parasympathetic balance. The power value of the peak on the fundamental frequency (*peakLF, peakHF*) is extracted too.

Combining the analysis in the two domains discussed above, the time-frequency analysis is performed. In this analysis the same parameters as in frequency domain were computed in ECG segments of a given time-length.

Regarding to the non-linear analysis, techniques such as: Poincaré graphs and entropy-based measures were extracted from HRV signal. Graphs of Poincaré are a type of graphics that try to represent the self-similarity of a signal. Graph plots the current interval vs. the previous intervals (Fishman et al., 2012). Normally fits an ellipse positioned on the axis identity and with center in the middle of RR intervals. The axes of the ellipse (SD1 for the vertical axis and SD2 to the horizontal axis) represent the variability in the short term (SD1) and long-term (SD2) variability (Brennan et al., 2002). Sample Entropy (sampen) is a factor that attempts to quantify the complexity or degree of new information generated (Richman and Moorman, 2011). The interpretation we can make of this parameter is basically that if entropy worth 0, then consecutive sequences are identical and the bigger its value most is the complexity of the analyzed signal.

**Figure 8:**
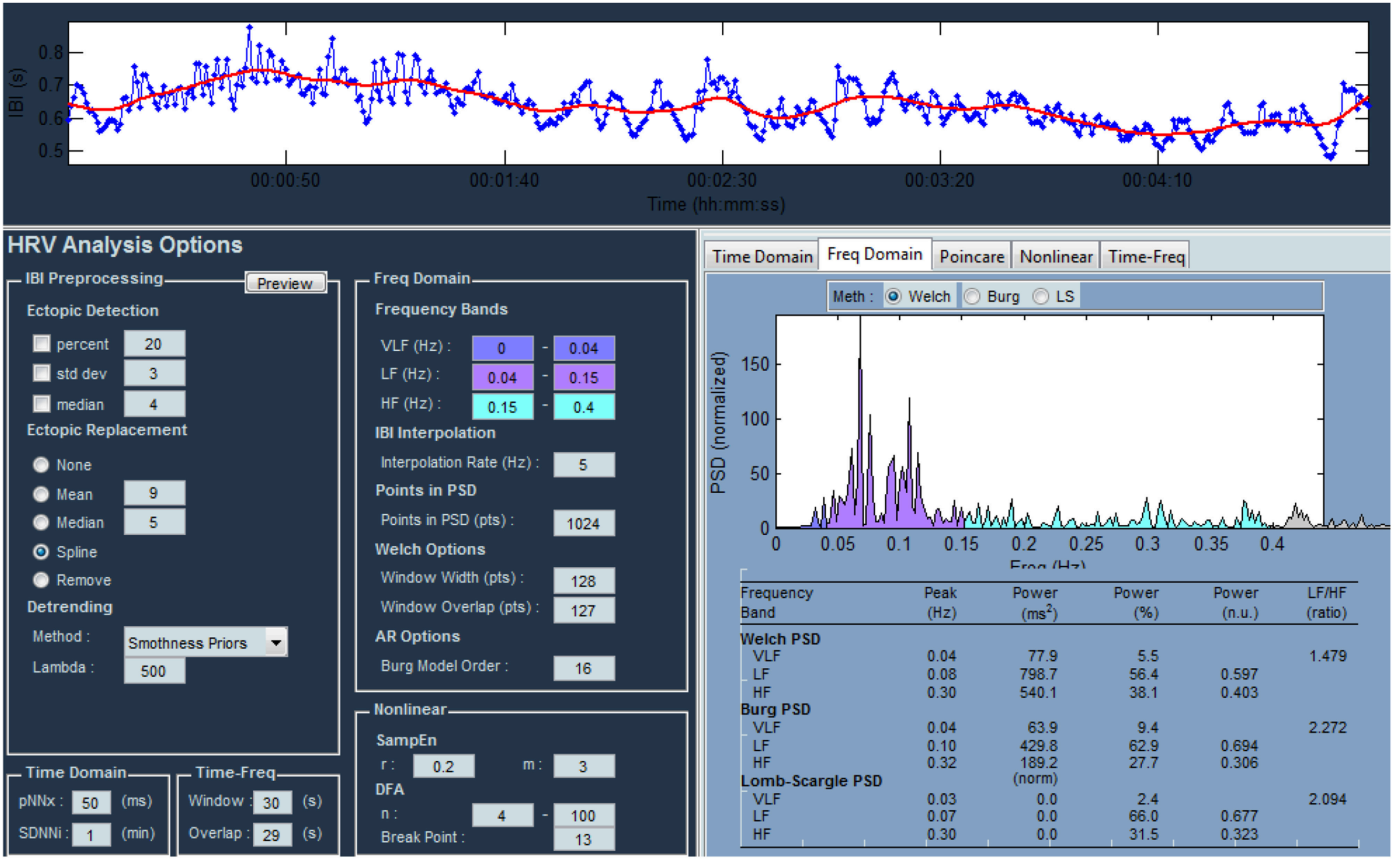
**Main window of the HRV analysis tool**.

For each stimulus and subject fifty-six parameters are computed by means of HRVAS tool (Ramshur, 2010; Guixeres et al., 2014) (Figure 8).

#### 2.4.3. GSR and RSP

**Figure 9:**
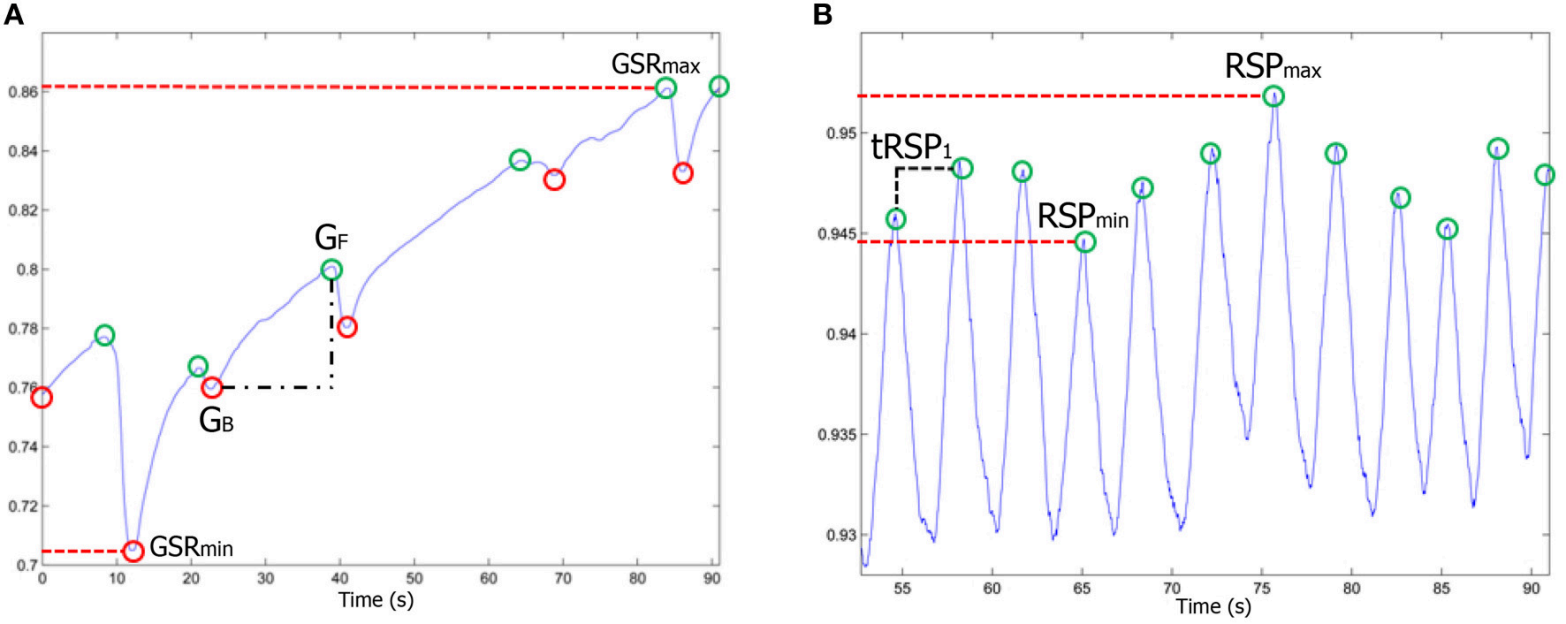
**(A)** GSR and **(B)** RSP physiological signals. The most representative parameters are highlighted.

Ten features were extracted from the Galvanic Skin Response signal (Figure 9A). The average, the variance and the standard deviation of the skin conductance along specific time periods under analysis (stimuli) was computed. In addition, the number of local maxima and minima and the mean conductivity difference (*G*_*F*_ − *G*_*B*_) for each consecutive pair of local minimum-maximum were calculated. For each stimulus, the global maximum *GSR*_*max*_ and minimum *GSR*_*min*_, the difference of them (*GSR*_*max*_ − *GSR*_*min*_) and the ratio between the number of maxima and stimuli duration (*peaks*∕*time*) were also extracted from GSR signals.

Regarding to RSP signal (Figure 9B), six physiological parameters during each stimulus were extracted. The respiratory rate, the average level of breathing, the longest and shortest time between consecutive breaths, the deep breathing (*RSP*_*max*_) and the shallow breathing (*RSP*_*min*_).

Table 2 shows a summary of the parameters extracted from each physiological signal. It is important to note that EEG parameters were calculated in each frequency band (excluding the emotional indexes calculated as described above).

**Table 2:** **Summary table showing all the parameters extracted from each biosignal used in this study**.

	**Category**	**Parameters used**
EEG features	Metrics based on Global Field Power	GFP Zscore log(Zscore)
	Emotional indexes	Interest Index (*II*) Memorization Index (*MI*) Pleasantness Index (*PI*)
	Frequency domain metrics	Power Spectral Density (PSD) Brainrate
ECG features	Time domain metrics	*MaxRR, MinRR* *MeanRR, MedianRR* *SDRR* *SDARR* *RMSSD* *RR*_50_ *p^RR^50*
	Frequency domain metrics	*aLF, aHF* *nLF, nHF* *pLF, pHF* *peakLF, peakHF*
	Time-frequency domain metrics	The same parameters extracted in frequency domain
	Non-linear analysis metrics	Poincaré Graphs (*SD1, SD2*) Entropy-based measures
GSR features	Time domain metrics	Average Variance Standard deviation Number of local minima Number of local maxima *Peaks*∕*Time* *G_F_* − *G_B_* *GSR_max_* and *GSR_min_* *GSR_max_* − *GSR_min_*
RSP features	Time domain metrics	Respiratory Rate Average level of Breathing Longest time between breaths Shortest time between breaths *RSP_max_* and *RSP_min_*

### 2.5. Classifiers

The different tested classifiers were Naive Bayes (John and Langley, 1995), Logistic Regression (Cessie and van Houwelingen, 1992), Multilayer Perceptron (Kohonen, 1988), Support Vector Machines (Chang and Lin, 2011), Linear Nearest Neighbor search (Weber et al., 1998), Random Forest (Breiman, 2001), AdaBoost (Freund and Schapire, 1996), Multiclass classifier (Bishop, 2006) and Bagging (Aslam et al., 2007). The used implementations of these classifiers are included in Weka (Hall et al., 2009; Witten et al., 2011), a broadly used data mining software and publicly available in Weka 3 (2009).

In order to reduce dimensionallity, AttributeSelectedClassifier also available in Weka was used (Witten and Frank, 2005). It is a meta-classifier that takes a search algorithm and evaluator similar to the base classifier. This makes the attribute selection process completely transparent and the base classifier receives only the reduced dataset. It works by finding a subset of features using the chosen feature selection method. It then uses this feature subset to train the specified classifier and output a classifier model. In addition, a wrapper (Kohavi and John, 1987) can be used within the AttributeSelectedClassifier as the feature selection method. The feature selection method is used to evaluate the accuracy of any feature subset. The wrapper can take any classifier and use it to perform feature selection. The advantage of using the wrapper is that the same machine learning algorithm can be used to evaluate the feature subset and also to train the final classifier, therefore expecting good results.

Once the features are extracted, the data of the dataset must be preprocessed before the classification step. In the pre-processing, two tasks are carried out: data normalization and data resampling. This is necessary because the range of values of raw data varies widely and the data set is clearly unbalanced and most machine learning algorithms would not work properly on that conditions. In this work, the method used for the normalization is to standardize all numeric attributes in the given dataset to have zero mean and unit variance and, for the resampling, the Synthetic Minority Oversampling TEchnique (SMOTE) (Chawla et al., 2002) was applied.

Classifiers were tested by means of 10-fold stratified cross-validation. In *k*-fold cross-validation, the original sample is randomly partitioned into *k* equal sized subsamples. Of the *k* subsamples, a single subsample is retained as the validation data for testing the model, and the remaining *k* − 1 subsamples are used as training data. The cross-validation process is then repeated *k* times (the folds), with each of the *k* subsamples used exactly once as the validation data. The *k* results from the folds are then averaged to produce a single estimation. In stratified k-fold cross-validation, the folds are selected so that the mean response value is approximately equal in all the folds (Schneider, J., 1997).

## 3. Results

In order to find which physiological signal was the best one, different datasets and combinations of them were used to perform the classification:

- EEG_GFP-ZSCORE: dataset with the GFP and Zcore metrics extracted from the EEG signal (18 features).
- EEG_PSD: dataset with the PSD metrics extracted from the EEG signal (72 features).
- EEG_IND: dataset with the Pleasantness, Memorization and Interest indexes' metrics extracted from the EEG signal (8 features).
- EEG_ALL: dataset with all the before mentioned metrics extracted from the EEG signal (98 features).
- HRV: all the metrics extracted from the HRV signal (56 features).
- GSR: all the metrics extracted from the GSR signal (10 features).
- RSP: all the metrics extracted from the Respiration signal (6 features).

Combination of signals:

- GSR + HRV: all the metrics extracted from GSR and HRV datasets (66 features).
- GSR + HRV + EEG_IND: all the metrics extracted from GSR, HRV and EEG_IND (Pleasantness, Memorization and Interest) datasets (74 features).
- GSR + HRV + EEG_ALL: all the metrics extracted from GSR, HRV and EEG (164 features).

Combination of signals using only features selected by AttributeSelectedClassifier:

- GSR_SEL + HRV_SEL: only selected metrics chosen by the best classifier with attribute selection from GSR and HRV datasets.
- GSR_SEL + HRV_SEL + EEG_IND_SEL: only selected metrics chosen by the best classifier with attribute selection from GSR, HRV and EEG_IND datasets.
- GSR_SEL + HRV_SEL + EEG_ALL_SEL: only selected metrics chosen by the best classifier with attribute selection from GSR, HRV and EEG_ALL datasets.

A list of the features included in each dataset can be found in Table 2. When combining signals, the instances (users) chosen to conform the dataset were those corresponding to the dataset of the signal with less instances, discarding all non-coincident instances from the other datasets.

Finally, the commercial under study, namely “The Date” from Heineken, was tested using the best classifier.

### 3.1. Analysis of the features extracted from the physiological signals

The classifiers used to test the datasets were three: Ranfom Forest (RF), Random Forest with attribute selection (ASC) and Random Forest with MultiClass Classifier (MCC) and Bagging (BAG). We chose Random Forest as starting point because it has proven to be a robust and efficient classifier independently of the dataset (Fernández-Delgado et al., 2014).

Datasets were previously balanced by means of SMOTE filter, and Standardized, to make all features of the same magnitude.

The classification of the datasets was performed in 3 rounds. In the first one, the goal was to evaluate each dataset individually. All datasets that obtained an accuracy of 75% or more were selected to participate in the second round (winning datasets). In the second round, combinations of winning datasets were evaluated. Lastly, in the third round, combinations of GSR and wining datasets were evaluated using only a subset of features (selected features). Selected features for each combination were chosen by applying to each combination of datasets the classifier which performed best with atribute selection.

Table 3 shows the classifier which performed best (among the three before mentioned) for each dataset and its accuracy.

**Table 3:** **Best result for each dataset**.

**Dataset**	**Algorithm**	**Positive**	**Neutral**	**Negative**	**Average**
EEG_ALL	MCC, BAG, RF	74.29	71.43	92.86	79.52
EEG_GFP-ZSCORE	MCC, BAG, RF	62.86	45.00	86.43	64.76
EEG_PSD	MCC, BAG, RF	57.71	65.14	94.29	72.38
**EEG_IND**	**MCC, BAG, RF**	**75.00**	**81.62**	**95.59**	**84.07**
RSP	RF	68.45	58.33	82.74	69.84
HRV	MCC, BAG, RF	75.46	75.46	88.34	79.75
GSR	ASC, RF	84.88	73.26	73.84	77.33
GSR + HRV	MCC, BAG, RF	75.00	78.57	86.43	80.00
GSR + HRV + EEG_ALL	MCC, BAG, RF	75.86	75.86	93.97	81.90
**GSR + HRV + EEG_IND**	**ASC, RF**	**79.31**	**90.52**	**91.38**	**87.07**
GSR_SEL	MCC, BAG, RF	89.29	85.00	80.71	85.00
**GSR_SEL + HRV_SEL**	**RF**	**95.00**	**89.29**	**78.57**	**87.62**
GSR_SEL + HRV_SEL + EEG_IND_SEL	MCC, BAG, RF	77.59	91.38	92.24	87.07

*Percentage of positive, neutral and negative ads correctly classified and average*.

*Bold values indicate the best performance obtained in each test and highlight the optimal combination of features for each dataset*.

As can be observed in Table 3, in the first round, the datasets that achieved an accuracy of 75% or greater were EEG_ALL, EEG_IND, HRV and GSR. The best accuracy was obtained with the EEG_IND dataset (84.07%) using Multi-Class, Bagging and Random Forest. Respiration metrics did not achieve a good classification accuracy (69.84%), and HRV accuracy (79.75%) and GSR accuracy (77.33%) were under the EEG accuracy (84.07%).

In the second round, the following combinations were tested: GSR + HRV, GSR + HRV + EEG_ALL and GSR + HRV + EEG_IND. In this case, the highest accuracy was obtained with Random Forest with atribute selection using a combination of all the features corresponding to GSR, HRV and EEG_IND metrics (87.07%). The other two combinations obtained 80.00 and 81.90%, respectively.

Lastly, in the third round, the datasets tested were: GSR, GSR + HRV and GSR + HRV + EEG_IND. These datasets had only the selected features by the atribute selection classifier. The highest accuracy obtained was with Random Forest using the selected attributes from the dataset combining GSR and HRV signals (87.62%), and the GSR dataset alone obtained 85.00% of accuracy using Multi-Class, Bagging and Random Forest.

The selected attributes for each dataset combination were:

- GSR: “Number of Peaks” and “Peaks/Time.”
- GSR + HRV: “Number of Peaks,” “Peaks/Time” and “t_SDANN”.
- GSR + HRV + EEG_IND dataset: “Number of Peaks,” “Peaks/Time,” “t_SDANN,” “mean_Theta,” “mean_Alfa,” “Index_Theta,” “Index_Beta,” “Peaks_Brand_Theta,” “Peaks_Theta,” “Peaks_Brand_Beta_Ext” and “Peaks_Beta_Ext.”

### 3.2. Comparison of classifiers

In the light of the results presented previously, the decision to test two more classifiers with the best 3 datasets was taken. The two new classifiers introduced were AdaBoost.M1 (AB) with Random Forest and Multi-Class with AdaBoost.M1 and Random Forest. Table 4 shows how these two new classifiers improved the accuracy by 2%, reaching the best result with the dataset conformed by the selected attributes from the GSR and HRV signals, obtaining 89.76% of accuracy.

**Table 4:** **Final results**.

**Dataset**	**Algorithm**	**Positive**	**Neutral**	**Negative**	**Average**
GSR_SEL	RF	90.00	83.57	79.29	84.29
	MCC,BAG,RF	89.29	85.00	80.71	85.00
	ASC,RF	90.00	83.57	79.29	84.29
	AB,RF	92.14	85.00	83.57	86.90
	MCC,AB,RF	91.43	85.00	81.43	85.95
**GSR_SEL + HRV_SEL**	RF	95.00	89.29	78.57	87.62
	MCC,BAG,RF	94.29	87.86	78.57	86.90
	ASC,RF	95.00	89.29	78.57	87.62
	AB,RF	95.00	91.43	80.71	89.05
	**MCC,AB,RF**	**95.00**	**91.43**	**82.86**	**89.76**
GSR_SEL + HRV_SEL + EEG_IND_SEL	RF	78.45	89.66	91.38	86.49
	MCC,BAG,RF	77.59	91.38	92.24	87.07
	ASC,RF	79.31	87.93	88.79	85.34
	AB,RF	80.17	87.93	91.38	86.49
	MCC,AB,RF	78.45	87.07	93.10	86.21

*Classifiers applied to the best datasets using only the features selected previously. Percentage of positive, neutral, negative and average results for the instances classified correctly*.

*Bold values indicate the best performance obtained in each test and highlight the optimal combination of features for each dataset*.

The configuration of this combination of classifiers is as follows: the MultiClass metaclassifier was employed using a 1-against-all strategy. The classifier used by MultiClass was AdaBoost. M1 with Random Forest as base classifier, 10 iterations, 100 as weight pruning threshold and reweighting. The RandomForest classifier was configured to generate 100 trees with unlimited depth and unlimited number of features to be used in random selection.

As a final test, the best dataset was classified with 11 more basic and advanced classifiers, but none was able to beat the current accuracy of 89.76% (Table 5).

**Table 5:** **Results for different classifiers applied to the best dataset: GSR (*Number of maxima, Peaks/Time*) + HRV (*t_SDANN*)**.

**Algorithm**	**Positive**	**Neutral**	**Negative**	**Average**
SVM	75.00	55.71	71.43	67.38
Multilayer Perceptron	75.00	49.29	67.14	63.81
Simple Logistic	75.00	36.43	85.71	65.71
Naive Bayes	75.00	20.00	75.00	56.67
Decision Table	75.00	22.86	83.57	60.48
Zero Rule	100.00	0.00	0.00	33.33
One Rule	57.86	56.43	60.00	58.10
Hoeffding Tree	74.29	52.14	47.86	58.10
Linear NN search	95.00	88.57	85.00	89.52
AdaBoostM1, Linear NN search	95.00	88.57	85.00	89.52
MultiClass, AdaBoostM1, Linear NN search	95.00	88.57	85.00	89.52
Random Forest	95.00	89.29	78.57	87.62
AdaBoostM1, Random Forest	95.00	91.43	80.71	89.05
**MultiClass, AdaBoostM1, Random Forest**	**95.00**	**91.43**	**82.86**	**89.76**

*The best accuracy using the following classifiers was obtained with the default parameters in the Weka software (Weka 3, 2009). Percentage of positive, neutral, negative and average results for the instances classified correctly*.

*Bold values indicate the best performance obtained in each test and which classifier provides the most accurate classification*.

Regarding to “The Date”, the commercial under study, the model obtained training the best dataset with the best classifier (i.e., Number of maxima and Peaks/Time from GSR, SDANN from HRV trained with MultiClass, AdaBoostM1 and RandomForest) was able to always classify “The Date” as positive.

## 4. Discussion

In this work we intended to build an algorithm able to classify commercials automatically. To achieve it, we built a model using the best possible data available and off-the-shelf algorithms.

Specifically, the main objective of this work was to find the most discriminatory or representative features that allowed to classify audiovisual content in 3 groups (positive, neutral and negative) with the highest possible accuracy. To accomplish this, we used EEG, GSR, HRV and respiration signals acquired from a group of 47 subjects while they were watching nine commercials. These commercials (excluding the ad under analysis) had been classified and labeled previously according to their Ace Score punctuation.

Tests performed show that the best classification was achieved using features extracted from GSR and HRV signals, namely “Number of maxima” and “Peaks/Time” from GSR, and “SDANN” from HRV, with an accuracy of 89.76%. On the other hand, the best accuracy with the EEG signal was 84.07%, attained with the dataset formed by the interest and pleasantness indexes. However, datasets with metrics extracted from EEG signal were the best in classifying only negative instances.

It is important to note that with just two features extracted from the GSR signal (“Number of maxima” and “Peaks/Time”) and a combined classifier consisting of Multi-Class, Bagging and Random Forest we were able to correctly classify 85% of the instances. This means that it is possible to obtain an accuracy very similar to the highest one—only 4.76% below of the highest accuracy obtained in this study and almost 1% above the best accuracy attained with EEG signals—with only two features from a single signal, which makes it very simple, usable and portable.

The implications of these results are that GSR and HRV signals provide more relevant information to classify an ad. That could mean that the Autonomic Nervous System is more useful for emotion classification than the Central Nervous System. This is supported by some other authors who state that GSR and HRV signals are able to accurately distinguish a user's emotion (Yoo et al., 2005; Li and h. Chen, 2006).

Validation of the model was performed using cross-validation in a first step and the commercial under evaluation (“The Date”) which was not used before to train or perform the cross-validation of the model was used in the test stage. Our model was able to classify this ad as positive.

The shortcomings of our method are mainly two. On the one hand, the attributes of the subjects could have been taken into account in the classification stage in order to evaluate commercials according to a specific population. On the other hand, a more comprehensive and exhaustive validation with more data could have been performed to get even more reliable results.

Regarding to the practical meaningfulness, these promising results could help to the development of an automatic system able to evaluate the quality of the commercials. This system could be helpful for the companies reducing the cost of their advertising design. Also, this kind of software would make possible the creation and evaluation of commercials focused in a particular audience.

In future works, voting majority could be used to improve the accuracy of each class independently, which could lead to better global results. In addition, other signals could be used as well to try to better discriminate among ads, such as Face Reader.

## 5. Conclusions

An automatic method able to classify commercials according its effectiveness is proposed in this paper. To achieve it, different features from physiological signals of 47 participants watching audiovisual contents were extracted and a model using the most discriminatory features was built. The different tests performed in this work show that the information provided by the GSR and HRV signals describe with an 89.76% of accuracy the effectiveness of the commercials. However, the negative commercials are better discriminated using the EEG features. These conclusions are promising in the audiovisual content evaluation field and might be an important direction for future research on commercial effectiveness.

## Author contributions

AC is the corresponding author. He is the specialist researcher in signal processing. AC investigated about the state of the art in signal preprocessing and feature extraction. He developed his own algorithms in order to preprocess the biosignals and extract the different parameters to the classification stage. FF is the specialist researcher in the classification stage. He used different advanced Machine Learning methods in order to classify commercials according to their effectiveness. FF has also assisted in the writing of this manuscript. VN is the director of this work. She guided to AC in order to investigate about preprocessing signal methods and to develop the home-made algorithms. VN reviewed the manuscript carefully and she gave to AC and FF helpful tips for the creation of the article. JG designed (with MA) the procedure of the experimental task and the biosignal acquisition. He participated in the revision of the final manuscript providing interesting comments and ideas. MA designed (with JG) the procedure of the experimental task and the biosignal acquisition. He participated in the revision of the final manuscript providing interesting comments and ideas. JA was responsible to the physiological signal acquisition. He reviewed the final manuscript.

### Conflict of interest statement

The authors declare that the research was conducted in the absence of any commercial or financial relationships that could be construed as a potential conflict of interest.
